# The significance of miR-124 in the diagnosis and prognosis of glioma: A systematic review

**DOI:** 10.1371/journal.pone.0312250

**Published:** 2024-11-01

**Authors:** Elham Ghasemi, Mahdieh Mondanizadeh, Amir Almasi-Hashiani, Elyar Mahboobi

**Affiliations:** 1 Department of Biotechnology and Molecular Medicine, School of Medicine, Arak University of Medical Sciences, Arak, Iran; 2 Student Research Committee, Arak University of Medical Sciences, Arak, Iran; 3 Department of Epidemiology, School of Health, Arak University of Medical Sciences, Arak, Markazi, Islamic Republic of Iran; 4 Traditional and Complementary Medicine Research Center, School of Medicine, Arak University of Medical Sciences, Arak, Iran; University of Nebraska Medical Center, UNITED STATES OF AMERICA

## Abstract

Glioma is a type of cancer that affects the central nervous system and necessitates a non-invasive diagnostic and prognostic assessment. MicroRNAs (miRNAs) play a crucial role in glioma and can provide valuable information about the prognosis of patients with this condition. MiR-124 is associated with molecules that play crucial roles in cellular processes, and any disruption in its expression can have a detrimental effect on cells, potentially leading to cancer. Therefore, miR-124 can be a valuable biomarker for diagnosis and prognosis in glioma. This review aims to highlight the role of miR-124 as a diagnostic and prognostic factor in glioma. To address this issue, we systemically reviewed and used various search strategies across three databases (PubMed, Web of Science and Scopus) and then yielded 3046 records from inception to September 2023. Records that did not meet our inclusion criteria were excluded. Following the screening process, our analysis included and summarized 13 eligible studies that not only measured miR-124 in serum, plasma, and tissue of glioma patients but also provided insights intomiR-124 as a prognostic and diagnostic biomarker. Thirteen studies were included for diagnostic accuracy, and five were considered for prognostic importance of miR-124. Based on our results, a single study showed an increase in miR-124 levels in exosomes obtained from patient serum, whereas the data from the 12 studies analyzed consistently pointed towards a reduction in miR-124 levels in various glioma samples. In conclusion, our findings suggest that miR-124 may be a useful diagnostic and prognostic biomarker in glioma. However, further investigations are required to draw more definitive conclusions.

## 1. Introduction

Glioma is a rapidly growing and aggressive brain tumor that is commonly found in the central nervous system. Despite the availability of treatments, the prognosis for glioma patients is grim, with an average life expectancy of around 5 years after the initial diagnosis [1]. Each year, the United States of America records over 22,000 new cases of glioma, with an estimated annual incidence rate of 7 cases per 100,000 people. As a result, glioma is considered the most prevalent primary tumor worldwide [2,3].

MiRNAs are small, single-stranded, non-coding RNA molecules that are approximately 19–25 nucleotides in length. They attach to the 3’ untranslated region (3’UTR) of messenger RNAs, and their attachment inhibits gene expression, ultimately resulting in reduced protein production [4,5]. Furthermore, miRNAs play a crucial role in regulating numerous cellular processes related to the development and advancement of cancer, such as tumor formation, invasion, DNA repair, and resistance to treatment. Their disruption in cancer suggests that they could be potential biomarkers for predicting and diagnosing cancer [6]. Certain miRNAs function as oncomiRs, being upregulated in malignancies, while others act as tumor suppressors, becoming downregulated in the majority of cancers [7,8].

MiR-124 is a type of miRNA that has been studied in the context of glioma [9]. The levels of miR-124 are significantly lower in glioma tissues compared to normal brain tissues, indicating that this miRNA may function as a tumor suppressor in the development of glioma [3]. After conducting research, it was found that miR-124 is composed of three separate members: miR-124-1, miR-124-2, and miR-124-3, each situated in different chromosomal regions. This ultimately results in elevated levels of miR-124 during the process of neural differentiation [10,11]. The expression of miR-124-3p is notably reduced in high-grade gliomas in comparison to low-grade gliomas, suggesting that the decrease in miR-124-3p levels could play a role in the advancement and increased severity of gliomas [12].

Our current understanding of the role of miR-124 in glioma is not fully elucidated. This review aims to provide a thorough analysis of the role of miR-124 in the diagnosis and prognosis of glioma, shedding light on its potential implications for understanding and managing this type of brain tumor.

## 2. Material and method

### 2.1 Eligibility criteria

In this comprehensive systematic review encompassed numerous studies highlighting the pivotal role of miR-124 as a prognostic and diagnostic marker for glioma patients, without limitations on publication date, population, or geographical location. This systematic review was carried out without consideration of the sex, race, age, or language of the patients. By The articles were categorized based on key outcomes like overall survival (OS), progression-free survival (PFS), and disease-free survival (DFS), and were then classified as articles related to diagnosis and prognosis. All methods were carried out in accordance with relevant guidelines and regulations. This study was approved by the local Medical Ethics Committee of Arak University of Medical Sciences with identification code: IR.ARAKMU.REC.1402.208.

### 2.2 Study design

The study designs of the papers involved were limited to case-control studies, providing prognosis and diagnosis value for miR-124. The study followed the guidelines of the Reporting Items for Systematic Reviews and Meta-Analyses (PRISMA) for its methodology [13]. The data was organized into two tables, one containing valuable information for prognosis and the other containing valuable information for diagnosis.

### 2.3 Search strategy

The articles were retrieved from electronic databases such as PubMed, Web of Science (Clarivate Analytics), and Scopus, with the search conducted from the earliest available date to September 2023. Google Scholar was used to search for gray literature. Additionally, the references of review articles were reviewed to ensure that no potentially relevant and suitable articles were overlooked. The key words and search strategy in Scopus, PubMed, and the Web of Science are included in Table 1.

**Table 1:** The search strategy in three international databases.

Databases	Query
**Web of science**	"Glioma" (Topic) or "Gliomas" (All Fields) or "Glial Cell Tumor" (All Fields) or "Mixed Glioma" (All Fields) or "Malignant Glioma" (All Fields) AND "MIRN124 microRNA" (Topic) or "miR-124" (All Fields) or "microRNA-124" (All Fields) or "hsa-miR-124" (All Fields) or "miRNA-124" (All Fields) or "miR124" (All Fields) 124
**PubMed**	("MIRN124 microRNA, human" [Supplementary Concept] OR "miR-124"[tw] OR "microRNA-124"[tw] OR "hsa-miR-124"[tw] OR "miRNA-124"[tw] OR "hsa-124"[tw] OR "mirR-124"[tw] OR "miR124"[tw]) AND ("Glioma"[Mesh] OR "Glioma"[tw] OR "Gliomas"[tw] OR "Glial Cell Tumor"[tw] OR "Mixed Glioma"[tw] OR "Malignant Glioma"[tw]) 119
**Scopus**	( (TITLE-ABS-KEY ("Glioma") OR TITLE-ABS-KEY ("Gliomas") OR TITLE-ABS-KEY ("Glial Cell Tumor") OR TITLE-ABS-KEY ("Mixed Glioma") OR TITLE-ABS-KEY ("Malignant Glioma") ) ) AND ( (TITLE-ABS-KEY ("MIRN124") OR TITLE-ABS-KEY ("microRNA") OR TITLE-ABS-KEY ("miR-124") OR TITLE-ABS-KEY ("microRNA-124") OR TITLE-ABS-KEY ("hsa-miR-124") OR TITLE-ABS-KEY ("miRNA-124") OR TITLE-ABS-KEY ("miR124") ) ) AND (LIMIT-TO (LANGUAGE, "English") ) AND (LIMIT-TO (DOCTYPE, "ar") ) 2,803

This systematic review used the PICO framework to conduct the search, where the population (P) was glioma patients, the intervention (I) and comparison (C) was between control and healthy tissue versus glioma tissue, and the outcome (O) was the examination of miR-124 expression between control tissue and glioma tissue.

### 2.4 Selection process

Two researchers (E.GH and E.M) independently and thoroughly reviewed articles based on title and abstract, then they screened articles based on full texts to selecting appropriate ones that met the following criteria for inclusion in this systematic review: (1) the prognostic and diagnostic role of miR-124 in glioma tissues and/or blood samples of glioma patients (2) original articles; (3) written in English; (4) no time limitation, spanning from the beginning to the day of search; (5) conducted on either a cell line or humans; and (6) studies that have PICO elements. Exclusion criteria were: (1) letters, case reports, reviews, conference abstracts; (2) non-English papers; (3) methodological studies; (4) studies not focused on glioma patients.

### 2.5 Data collection process

Data that met the inclusion criteria were compiled into a personalized excel spreadsheet database by one investigator (E.G), and then these entries were verified by another investigator (E.M). The extracted information for each study included: the first author`s name; publication year; country; sample type; sample size; method; miR-124 expression; gender; mean age; disease stage; parameters significantly associated with prognosis; survival analysis and Newcastle-Ottawa Scale (NOS) score, which is a tool used for assessing the quality of non-randomized studies.

### 2.6 Study risk of bias assessment

Quality assessment was accomplished through NOS for the assessment of the quality and to evaluate the potential risk of bias [14]. The entered articles are evaluated in terms of quality, considering that the type of articles entered are observational (case–control); this checklist is used. The articles are classified into three groups according to their quality. Based on this checklist, articles with a score of 0–3 are considered weak, articles with a score of 3–6 are considered average, and articles with a score of 6–9 are considered high quality.

## 3. Result

### 3.1. Study selection

**Fig 1:**
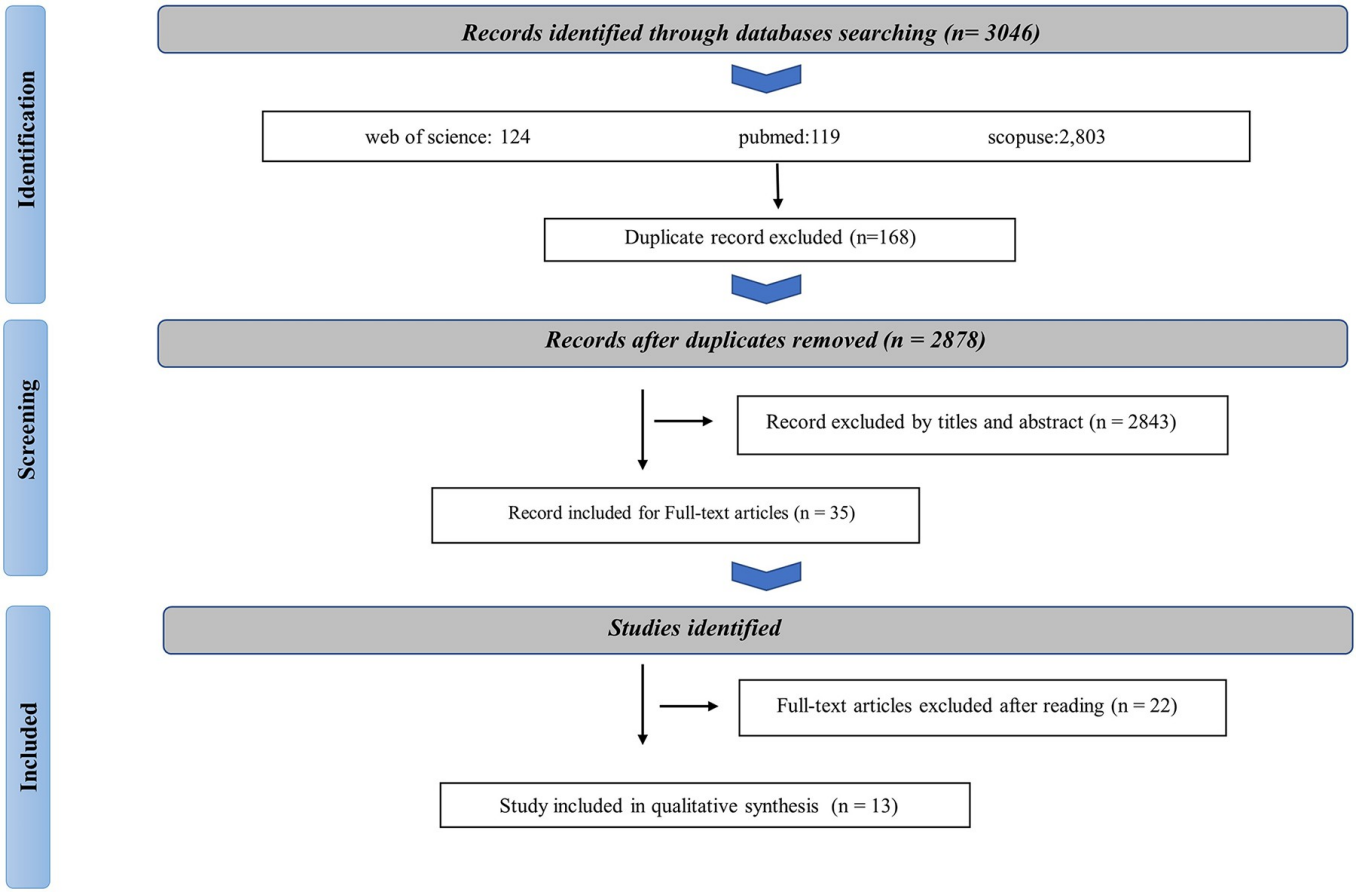
Screening flowchart for selecting articles in this systematic review.

Initially, a search at beginning offered 3046 records which were identified as valid databases. PubMed provided 119 references, Web of Science provided 124 references and Scopus provided 2,803 references. After eliminating 168 duplicates, 2843 articles were excluded based on irrelevant titles and abstracts. Subsequently, 35 full-text articles were studied, and 22 articles were excluded due to reasons such as not meeting inclusion criteria or lacking available prognostic-related data. The list of excluded studies with reasons were presented in Appendix 1. Ultimately, 13 eligible articles were involved in the study (Fig 1).

These selected studies focus on evaluating miR-124 as a diagnostic and prognostic factor in tissue, plasma, and serum. This systematic review covers studies published between 2011 and 2021 with case sample sizes ranging from 16 to 141 (total: 633) and control sample sizes ranging from 4 to 40 (total: 136). It is important to note that two studies did not report their control sample size, and 2 studies did not give information about case sample size. Among the 13 articles, three of them cited miR-124 as a diagnostic factor accompanied by others miRNAs, while 10 explicitly focused on miR-124. Additionally, 10 papers did not indicate the mean age of the patients. All the papers used the qReal-time PCR technique to measure miR-124 expression, with only one paper examining miR-124 expression in plasma, two papers investigating miR-124 expression in patient serum, and the remaining 10 articles investigating miR-124 in tissue.

### 3.2 Results of individual studies

#### 3.2.1 Diagnostic value of miR-124

In 12 out of 13 studies, it was found that miR-124 is decreased in both glioma tissue and serum, indicating its potential as a diagnostic marker. Conversely, one study among the 13 indicated that miR-124 is increased in exosomes isolated from the serum of glioma patients.

Many of these studies not only perceive miR-124 as a diagnostic factor but also note its correlation with numerous molecules. For instance, Wei-hua Zhao et al. displayed that miR-124 negatively regulates PPP1R13L (an inhibitory member of the apoptosis-stimulating protein of p53 family whit a role in growth, metastasis, and apoptosis) expression and also showed miR-124 is downregulated in glioma tissue [15]. Shuai Xiao et al. demonstrated that miR-124 negatively regulates TP73-AS1 (a lncRNA that adjust apoptosis by p53-dependent antiapoptotic gene regulation) and iASPP (inhibitor of apoptosis-stimulating protein of p53) and its down regulation in glioma tissue [16]. Additionally, Qiang Chen et al. demonstrated in 2013 that miR-124-5p binds to the 3’ UTR of LAMB1 (which codes the b1 chain of laminin-8 in glioma cells lines) to control its expression. The upregulation of LAMB1 expression was strongly associated with the downregulation of miR-124-5p. miR-124-5p suppressed LAMB1 protein expression. Restoring miR-124-5p expression inhibited glioma growth by suppressing angiogenesis, similar to the effects observed upon LAMB1 knockdown [17]. In 2014, Zhumei Shi et al. verified that miR-124 directly targets R-Ras (related Ras viral oncogene homolog) and N-Ras(neuroblastoma Ras viral oncogene homolog), and also inhibits the Akt and Raf/ERK1/2 signaling pathways and mentioned miR-124 downregulation in glioma tissue [18]. In 2013, Liwen An et al. found a negative association between miR-124 and ROCK 1 (rho-associated coiled-coil containing protein kinase 1), which is cell mobility-related gene and also mentioned miR-124 expression decreased in glioma tissue [19]. Qiaoli Wu et al. in 2018 used several techniques to prove a connection between miR-124 and Ephrin type-A receptor 2 (EphA2) and role of miR-124 downregulation in glioma tissue [20]. In 2017, Xiangrong Liu et al. stated that iASPP is identified as one of the direct targets of miR-124 and also considered miR-124 expression decreased in glioma tissue [21]. Alenka Matjašič et al. revealed that primary and secondary glioblastomas (GBMs) had significantly reduced expression of miR-124a and RNCR3 (retinal noncoding RNA 3), and a positive correlation between the expression of these two non-coding RNAs. The decreased expression of miR-124a could be a result of the reduced expression of its precursor, RNCR3 [22]. In 2010, Adam Fowler et al. proposed that there is a potential correlation between the expression of miR-124a and IQGAP1 (IQ motif containing GTPase activating protein 1), LAMC1(laminin c1), and ITGB1 (integrin b1) [23]. In 2016, Wanghao Chen et al. proposed that the microRNA expression pattern may serve as a biomarker for the prognosis and prediction of glioblastoma recurrence. By analyzing microarray gene expression data and clinical information, they identified 7 microRNAs including miR-124a. They found the expression of miR-124a was down-regulated in GBM tissues [24]. In 2021, Debora Olioso et al. conducted a study on the levels of miR-21, miR-222, and miR-124-3p in the serum of high grade glioma patients [25]. Additionally, the study by L.-Q. WANG et al. suggests that the increased expression of serum exosomal miR-21, miR-222, and miR-124-3p during post-operative follow-up is associated with the progression of high-grade gliomas. These findings support the use of exosomal miR-21, miR-222, and miR-124-3p as complementary molecular biomarkers for the clinical evaluation of tumor relapse during post-surgical longitudinal monitoring of high-grade glioma patients. [26]. In 2017, Alessandra Santangelo et al. also examined that the expression levels of miR-21, miR-222 and miR-124-3p in serum exosomes were significantly higher in patients with high grade gliomas compared to those with low grade gliomas and healthy controls, and sharply decreased after surgery. Analysis of these miRNAs in serum exosomes can provide a minimally invasive and innovative tool to help diagnose gliomas at onset, predict their grade, and detect non-glial metastases before surgery [27] (**Table 2**).

**Table 2:** Investigating the potential of miR-124 expression as a diagnostic factor.

First author	Year	Country	Study design	Sample Type	sample size (control)	Sample Size (case)	Gender	Range age (year)	Mean age (year)	Glioma type	miR-124 Expression	Analysis method	NOS	Data extractor	Date of extraction
Wei-hua Zhao[15]	2013	China	case-control	Tissue	NR	9	NR	NR	NR	Glioblastoma	miR-124 is downregulated, negatively regulates PPP1R13L.	Real time RT-PCR, luciferase	5	EG and EM	Feb, 2024
Shuai Xiao [16]	2018	China	case-control	Tissue	NR	30	NR	NR	NR	NR	miR-124 expression is decreased, TP73-AS1 negatively regulates miR-124.	real-time PCR- luciferase	4	EG and EM	Feb, 2024
Qiang Chen [17]	2013	China	case-control	Tissue	NR	NR	NR	NR	NR	All type	The upregulation of LAMB1 expression was highly correlated with the downregulation of miR-124-5p. LAMB1 protein expression was suppressed by miR-124-5p. The restoration of miR-124-5p expression suppressed glioma growth by inhibiting angiogenesis, effects that were also observed upon LAMB1 knockdown.	Real time qPCR, luciferase	4	EG and EM	Feb, 2024
Zhumei Shi [18]	2014	China	case-control	Tissue	6	24	NR	NR	NR	Glioblastoma, astrocytoma, anaplastic astrocytoma	miR-124 is downregulated in glioma, miR-124 negatively regulates R-Ras and N-Ras expression, regulates Ras/Akt and Ras/Raf/ERK1/2 pathway.	qRT-PCR, Dual-luciferase	7	EG and EM	Feb, 2024
Liwen An [19]	2013	China	case-control	Tissue	NR	16	NR	NR	NR	All type	miR-124 is downregulated in glioma, Interacts with the 3 UTR Region of ROCK 1.	Real time qPCR, luciferase	5	EG and EM	Feb, 2024
Qiaoli Wu [20]	2018	China	case-control	Tissue	20	20	NR	NR	NR	NR	miR-124 is decreased, negatively interact with EphA2.	Real time PCR, RT-PCR, luciferase	5	EG and EM	Feb, 2024
Xiangrong Liu [21]	2017	China	case-control	Tissue	14	66	Both	NR	NR	Diffuse astrocytoma, anaplastic astrocytoma, glioblastoma multiform	miR-124 is downregulated, interacts with ASPP, (CDK)4, CDK6 and cyclin D.	qRT-PCR	6	EG and EM	Feb, 2024
Alenka Matjašič [22]	2017	Slovenia	case-control	Tissue	NR	64	Both	51.8	NR	Astrocytoma, Oligodendroglia, Oligoastrocytoma	primary and secondary glioblastomas had significantly reduced expression of miR-124a and RNCR3, and a positive correlation between the expression of these two non-coding RNAs. The decreased expression of miR-124a could be a result of the reduced expression of its precursor, RNCR3.	qRT-PCR	7	EG and EM	Feb, 2024
Adam Fowler [23]	2010	Australia	case-control	Tissue	4	117	Both	63.86 58.56	27–84 33–85	Glioblastoma	miR-124a is down-regulated, regulates IQGAP1, LAMC1 and ITGB1.	RT-qPCR	7	EG and EM	Feb, 2024
Wanghao Chen [24]	2016	China	case-control	Tissue	10	34	Both	NR	NR	Glioblastoma, Astrocytoma	The microRNA expression pattern, including the downregulation of miR-124a, may serve as a biomarker for the prognosis and prediction of glioblastoma recurrence.	RT-PCR	7	EG and EM	Feb, 2024
Debora Olioso [25]	2021	Italy	case-control	serum	NR	57	Both	63	23–88	Glioblastoma, Anaplastic Astrocytoma	miR-21, -222 and -124-3p in serum exosomes examined, miR-124-3p is downregulated.	Real time qPCR	6	EG and EM	Feb, 2024
L.-Q. Wang [26]	2019	China	case-control	plasma	40	64	Both	NR	NR	All type	the study suggests that the expression of exosomal miR-21, miR-222 and miR-124-3p can be used as complementary molecular biomarkers to clinically evaluate tumor relapse during post-surgical longitudinal monitoring of high-grade glioma patients.	qRT-PCR	7	EG and EM	Feb, 2024
Alessandra Santangelo [27]	2017	Italy	case-control	Serum	30	111	Both	NR	41–61	All type	The expression levels of miR-21, miR-222 and miR-124-3p in serum exosomes were significantly higher in high grade gliomas compared to low grade gliomas and healthy controls, and decreased after surgery. Analysis of these miRNAs in serum exosomes can provide a minimally invasive tool to help diagnose and grade gliomas, and detect non-glial metastases before surgery.	Real time qPCR	7	EG and EM	Feb, 2024

NR: Not reported, GBM: Glioblastoma, LGG: Low grade glioma, qPCR: Quantitative PCR, qRT-PCR: Quantities reverse transcriptase, NOS: Newcastle-Ottawa Scale

#### 3.2.2 prognostic value of miR-124

Out of the 13 studies, four of them evaluated miR-124 as a prognostic indicator. All of these studies established that the level of miR-124 expression can be utilized as a prognostic indicator in glioma patients with varying grades [23–26]. Debora Olioso conducted a Kaplan-Meier analysis which revealed that in patients with low miR-124-3p expression, treatment with TMZ resulted in PFS increased to about 10.4 a months and 23.9 month increase in OS [25]. Adam Fowler conducted Kaplan–Meier survival curve analysis and apprehended a correlation between miR-124a and overall survival. Out of 117 patients, 52 showed higher expression of miR-124a compared to the average expression. The median survival for these patients was 13.9 months, while patients with low miR-124a expression levels had a median OS of 13.3 months [23]. L.-Q. Wang conducted Kaplan-Meier analysis to assess the OS and DFS of patients. They discovered that individuals with low plasma miR-124 expression had remarkably shorter overall survival, with a hazard ratio of 0.857 for OS and 0.851 for DFS [26]. Wanghao Chen conducted Kaplan-Meier analysis to study the OS and DFS in seven miRNAs. The DFS for miR124a was found to be 0.124, while the OS was 0.13 [24] (**Table 3**).

**Table 3:** MiR-124 expression as a prognosis factor.

First author	year	location	Result	ref
**Adam Fowler**	2011	Australia	The expression of miR-124 was examined in 117 patients with high-grade and low-grade glioma. Among them, 52 patients (44%) exhibited a high level of miR-124a, with a median survival of 13.9 months. In contrast, patients with low miR-124a levels showed a median survival of 13.3 months. (p = 0.481)	[23]
**L.-Q. WANG**	2019	China	miR-124 was examined in all types of glioma, and the ROC curve (AUC) showed a value of 0.851 for DFS and 0.857 for OS. Patients with low levels of plasma miR-124 had notably shorter DFS (p < 0.001), and those with low levels of plasma miR-124 had significantly shorter OS (p < 0.001).	[26]
**Wanghao Chen**	2016	China	The Kaplan-Meier survival analysis using the log-rank test showed that the disease-free survival (DFS) was 0.124 and the overall survival (OS) was 0.13.	[24]
**Debora Olioso**	2021	Italy	The median PFS was 10.4 months, and OS was 23.9 months for patients with low miR-124-3p expression (p-values were 0.048 and 0.031, respectively).	[25]

OS: Overall Survival, DFS: Disease Free Survive.

### 3.3 Risk of bias in studies

In terms of quality, all included studies were evaluated by the Newcastle-Ottawa Scale (NOS) checklist. The study conducted by Alessandra Santangelo [27], Zhumei Shi [18], L.-Q. Wang [26], Alenka Matjašič [22], Adam Fowler [23], and Wanghao Chen [24] received the highest score (score 7), whereas the studies conducted by Debora Olioso [25] and Xiangrong Liu [21] got a score of 6. Wei-hua [15], Liwen An [19], and Qiaoli Wu [20] all achieved a score of 5, while the study conducted by Qiang Chen [17] and Shuai Xiao [16] received the lowest quality score of 4 (Table 4).

**Table 4:** The results of studies quality score based on NOS checklist.

Questions/articles	Wei-hua Zhao[15]	Shuai Xiao [16]	Qiang Chen [17]	Zhumei Shi [18]	Liwen An [19]	Qiaoli Wu [20]	Xiangrong Liu [21]	Alenka Matjašič [22]	Adam Fowler [23]	Wanghao Chen [24]	Debora Olioso [25]	L.-Q. Wang [26]	Alessandra Santangelo [27]
**Case definition**	*			*		*	*	*	*	*	*	*	*
**Representativeness of the cases**				*		*		*	*	*	*	*	*
**Selection of Controls**													
**Definition of Controls**				*	*		*	*	*	*		*	*
**Comparability of cases and controls**	*	*	*	*	*	*	*	*	*	*	*	*	*
**Ascertainment of exposure**	*	*	*	*	*	*	*	*	*	*	*	*	*
**Same method of ascertainment for cases and controls**	*	*	*	*	*		*	*	*	*	*	*	*
**Non-Response rate**	*	*	*	*	*	*	*	*	*	*	*	*	*
**Final score**	5	4	4	7	5	5	6	7	7	7	6	7	7

## 4. Discussion

Compelling research has demonstrated the significant clinical importance of miRNAs as prognostic and diagnostic biomarkers in glioma, highlighting their intricate role [28–30]. miR-124 plays a crucial role in the development of tumors, as well as in the invasion and spread of glioma [31,32]. We have agreed to conduct a thorough systematic review that examines the significance of miR-124 in serving as a diagnostic and prognostic biomarker. With the exception of one paper indicating an upregulation of miR-124 in glioma, all the research demonstrated a downregulation of miR-124 in glioma. Nevertheless, number of 12 studies reviewed in this systematic analysis confirmed the significance of miR-124 as a diagnostic biomarker, and four of them also emphasized its credibility as a prognostic indicator for glioma.

In 2013, Zhonghua Lv work illuminated the crucial role of Grb2/Sos1 in the Receptor Tyrosine kinase (RTKs) pathway. The findings indicated that miR-124 directly targets the 3’UTR of SOS1(Son of sevenless 1), converting Ras-GDP into Ras-GTP, thereby activating the RTKs pathway, which plays a role in growth, proliferation, and apoptosis. Inhibition of SOS1 expression leads to the suppression of growth in the U87 cell line. Additionally, the study revealed that miR-124 exerts a negative regulatory influence on the mitogen-activated protein kinase (MAPK) pathway, which is involved in cell proliferation [33]. In 2014, Shao-Hua Lu noted a downregulation of miR-124a in both glioma tissue and glioma cell lines. Through their research, it was observed that miR-124a negatively regulates IQGAP1 (IQ motif containing GTPase activating protein 1), a protein involved in cellular processes, leading to the suppression of β-catenin and cyclin D1. This ultimately results in increased proliferation and invasion in glioma cells [34]. In 2015, Jia-JuNn Cai and Zeng-Xin Qi discovered that there is a notable decrease in miR-124 expression in glioma tissue. This decrease was found to correspond with an elevation increase in Calpain small subunit 1 (Capn4) levels. This molecule plays a critical role in regulating invasion and migration in glioma tissue, so this pivotal imbalance sheds light on the invasion and migration in glioma [35]. In a study conducted in 2016, Weihao Li revealed Weihao Li demonstrated that miR-124 binds to the 3′-UTR of signal transducer and activator of transcription 3 (STAT3) that regulates the expression of target genes important in cell cycle progression, apoptosis and mitosis, and can act as a negative regulator. Downregulation of miR-124 led to elevated levels of STAT3 in glioma cells. Conversely, upregulation of miR-124 demonstrated a remarkable impact by inhibiting cell proliferation and increasing apoptosis in U87 and U251 glioma cells [36]. In 2017, Zhonghua Lv demonstrated that miR-124 is initially downregulated in glioma tissue and cells. Lv’s research further revealedthat the restoration of miR-124 leads to the inhibition of proliferation and invasion in glioma cells. This inhibition was achieved through binding to the 3′-UTR of Smad2 (suppressor of mothers against decapentaplegic) which monitors normal cell growth, and Smads have a role in the transforming growth factor-beta (TGF-β) signaling pathway [37]. In 2018, Lifei Luo observed a decrease in the level of miR-124-3p in glioma tissue and glioma cell lines. Additionally, through experimental transfection, Luo demonstrated that the transfection of miR-124-3p led to a reduction in Fos-related antigen-2 (Fra-2) by binding to its 3′-UTR. Fra-2 belongs to AP-1 transcription factor family and is related to cell-cycle drivers(Cyclin D1 and Cyclin E1), an invasion-associated gene (MMP9), the mesenchymal marker (Vimentin), and the induction of the epithelial marker (E-cadherin), ultimately inhibiting the proliferation, migration, invasion, and tumorigenicity of glioma cells [12]. In 2018, Guilong Zhang demonstrated that miR-124-3p binds to the 3’ UTR of neuropilin-1 (NRP-1) and functions as a negative regulator. Downregulation of miR-124-3p led to an increase in the level of NRP-1, which in turn played a crucial role in tumorigenesis, invasion, and angiogenesis by interacting with key receptors like class 3semaphorins (SEMA3), vascular endothelial growth factor (VEGF) or transforming growth factor b1 (TGF-B1), ultimately resulting in the repression of tumor angiogenesis and apoptosis in glioma cells, as well as the inhibition of glioma growth upon overexpression of miR-124-3p [38]. In 2019, Danni Deng demonstrated that the increase in p62 (an autophagy adaptor) expression, also known as sequestosome-1 (SQSTM1), promotes the advancement of glioma cell lines. Conversely, miR-124-3p negatively regulates p62, and a decrease in miR-124-3p leads to elevated p62 levels [30]. Multiple studies have consistently demonstrated a decrease in miR-124 levels in glioma tissue and cell lines. After conducting thorough screening and analysis of various research papers, it has been verified that the expression of miR-124 is decreased in glioma patient tissue, cell lines, serum, and plasma. Additionally, the reduction of miR-124 is linked to the stage of glioma, with more aggressive gliomas exhibiting a more significant decrease in miR-124 levels.

This study demonstrates numerous strong points and advantages, such as: 1) a comprehensive research approach that involved searching in three reputable databases and ultimately resulted in incorporation of 13 valuable articles, 2) no time limitations were imposed on the research, 3) inclusion of both human studies and cell line studies in the review, 4) adherence to the PRISMA checklist for all research protocol steps, 5) the study is based on the PICO framework, and 6) The consistent finding of lower miR-124 levels in cancerous tissues across various cancer types suggests it could be useful as both a prognostic marker and a diagnostic tool.

The existing review has several limitations: 1) This review is limited by the fact that it only includes studies conducted in the English language (language restriction). 2) Many studies lacked crucial data such as OS, disease-free survival DFS, and prognostic information, making it difficult to conduct a meta-analysis. 3) Some studies lack adequate reporting of important factors like mean age, sample size of the control group, gender, and glioma stage, resulting in heterogeneity.

We strongly recommend: 1) Further research is urgently needed to explore the prognostic and diagnostic significance of miRNAs, especially miR-124, 2) Papers should provide detailed statistical measures like OS, PFS, DFS, and others for potential use in future systematic reviews and meta-analyses. 3) Reporting the average age, age range and gender of subjects, as well as specifying the level of miRNA expression based on cancer stage, is essential. 4) study on isocitrate dehydrogenase (IDH) status, 1p19q codeletion or some considerable information would be valuable.

Inconclusion, the evidence suggests that miR-124 plays a significant role in regulating various biological processes and is implicated in the pathogenesis of multiple cancers. Its expression levels in normal and cancerous tissues provide insights into its potential as a diagnostic biomarker. miR-124 is predominantly expressed in the brain but also found in other normal tissues, participating in neurogenesis and neuronal differentiation. In general, miR-124 levels are higher in healthy tissues compared to cancerous tissues, indicating its potential role as a tumor suppressor. The consistent pattern of reduced miR-124 levels in cancerous tissues across multiple cancer types suggests it could serve as a prognostic marker and diagnostic tool. The ability to detect decreased miR-124 levels in early-stage cancers may facilitate earlier diagnosis and intervention, making it a valuable candidate for further research in cancer diagnostics. Our analysis of 12 papers revealed a consensus that miR-124 is downregulated in glioma tissue, cell lines, serum, and plasma, with one study presenting conflicting results. Furthermore, four studies indicated that miR-124 is linked to overall survival, disease-free survival, or prognosis in glioma patients.

## Supporting information

S1 Checklist PRISMA 2020 checklist. (DOCX)

S1 Appendix List of excluded studies. (DOCX)
