# Supplementary material for: The significance of miR-124 in the diagnosis and prognosis of glioma: A systematic review
Source: PLoS One. 2024 Nov 1;19(11):e0312250. doi: 10.1371/journal.pone.0312250 (PMC11530070; doi:10.1371/journal.pone.0312250)
Supplement: S1 Appendix — (DOCX) [file pone.0312250.s002.docx]

**Appendix 1**. The list of excluded studies with reasons

|  | Articles Title | Author | year | country | Exclusion reason | Data  extractor | Date of  extraction | ref |
| --- | --- | --- | --- | --- | --- | --- | --- | --- |
| 1 | Grade-Specific Expression Profiles of miRNAs/mRNAs and Docking Study in Human Grade I–III Astrocytomas | Dan Li | 2011 | China | Lacke of available diagnostic and prognostic value | EG and EM | February 2024 | (1) |
| 2 | Reduced Expression of Brain-Enriched microRNAs in Glioblastoma as Permits Targeted Regulation of a Cell Death Gene | Rebecca L. Skalsky | 2011 | United States of America | Lacke of available diagnostic and prognostic value | EG and EM | February 2024 | (2) |
| 3 | Associations between microRNA expression  and mesenchymal marker gene expression  in glioblastoma | Xinlong Ma | 2012 | Japan | Lacke of available diagnostic and prognostic value | EG and EM | February 2024 | (3) |
| 4 | miR-124 inhibits the growth of glioblastoma  through the downregulation of SOS1 | ZHONGHUA LV | 2013 | China | Not meeting inclusion criteria | EG and EM | February 2024 | (4) |
| 5 | miR-124a restoration inhibits glioma cell proliferation and invasion by suppressing IQGAP1 and β-catenin | SHAO-HUA LU | 2014 | China | Lacke of available diagnostic and prognostic value | EG and EM | February 2024 | (5) |
| 6 | Prediction of clinical outcome in glioblastoma using a biologically relevant nine-microRNA signature | Josie Hayes | 2014 | USA | Lacke of available diagnostic and prognostic value | EG and EM | February 2024 | (6) |
| 7 | miR-124 suppresses the migration and invasion  of glioma cells in vitro via Capn4 | JIA-JUN CAI | 2015 | China | Lacke of available diagnostic and prognostic value | EG and EM | February 2024 | (7) |
| 8 | miR-124 Acts as a Tumor Suppressor in Glioblastoma via the Inhibition of Signal Transducer and Activator of Transcription 3 | Weihao Li | 2016 | China | Lacke of available diagnostic and prognostic value | EG and EM | February 2024 | (8) |
| 9 | MiR-124 inhibits cell proliferation, invasion,  and migration in glioma by targeting Smad2 | Zhonghua Lv | 2017 | China | Lacke of available diagnostic and prognostic value | EG and EM | February 2024 | (9) |
| 10 | Studying the MicroRNA role as a survival predictor and revealing its part in malignancy level determination in patients with supratentorial gliomas of brain | E. V. Stupak | 2017 | Russia | Lacke of available diagnostic and prognostic value | EG and EM | February 2024 | (10) |
| 11 | Identification of microRNAs associated with glioma diagnosis and prognosis | Xinyun Ye | 2017 | China | Not meeting inclusion criteria | EG and EM | February 2024 | (11) |
| 12 | MiR-124-3p suppresses glioma aggressiveness via targeting of Fra-2 | Lifei Luo | 2018 | China | Not meeting inclusion criteria | EG and EM | February 2024 | (12) |
| 13 | miRNA-124-3p/neuropilin-1(NRP-1) axis plays an important role in mediating glioblastoma growth and angiogenesis | Guilong Zhang | 2018 | China | Lacke of available diagnostic and prognostic value | EG and EM | February 2024 | (13) |
| 14 | p62 acts as an oncogene and is targeted  by miR-124-3p in glioma | Danni Deng | 2019 | 2019 | Not meeting inclusion criteria | EG and EM | February 2024 | (14) |
| 15 | MiR-124 inhibits malignant biological behaviors  of glioma cells by targeting SDCBP | Banyou Ma | 2019 | China | Lacke of available diagnostic and prognostic value | EG and EM | February 2024 | (15) |
| 16 | Prognostic Significance of MicroRNAs in Glioma:  A Systematic Review and Meta-Analysis | Yanming Zhang | 2019 | China | Not meeting inclusion criteria | EG and EM | February 2024 | (16) |
| 17 | MicroRNA-124-3p regulates cell proliferation,  invasion, apoptosis, and bioenergetics by targeting PIM1 in astrocytoma | Danni Deng | 2016 | China | Lacke of available diagnostic and prognostic value | EG and EM | February 2024 | (17) |
| 18 | Hypoxia-induced lncRNA PDIA3P1 promotes  mesenchymal transition via sponging of  miR-124-3p in glioma | Shaobo Wang | 2020 | China | Lacke of available diagnostic and prognostic value | EG and EM | February 2024 | (18) |
| 19 | miR‑124‑3p inhibits the viability and motility  of glioblastoma multiforme by targeting RhoG | SHAN cAI | 2021 | China | Lacke of available diagnostic and prognostic value | EG and EM | February 2024 | (19) |
| 20 | Long Non-coding RNA MALAT1 Upregulates ZEB2 Expression to Promote Malignant Progression of Glioma by Attenuating miR-124 | Hongyu Cheng | 2020 | China | Lacke of available diagnostic and prognostic value | EG and EM | February 2024 | (20) |
| 21 | MicroRNA profiling in the malignant progression of gliomas | E. V. Stupak | 2016 | Russia | Lacke of available diagnostic and prognostic value | EG and EM | February 2024 | (21) |
| 22 | Inhibition of EMMPRIN by microRNA‑124 suppresses the growth, invasion and tumorigenicity of gliomas | YANBIN SONG | 2021 | China | Lacke of available diagnostic and prognostic value | EG and EM | February 2024 | (22) |

**Reference**

1. Li D, Chen P, Li X-Y, Zhang L-Y, Xiong W, Zhou M, et al. Grade-specific expression profiles of miRNAs/mRNAs and docking study in human grade I–III astrocytomas. Omics: a journal of integrative biology. 2011;15(10):673-82.

2. Skalsky RL, Cullen BR. Reduced expression of brain-enriched microRNAs in glioblastomas permits targeted regulation of a cell death gene. PloS one. 2011;6(9):e24248.

3. Ma X, Yoshimoto K, Guan Y, Hata N, Mizoguchi M, Sagata N, et al. Associations between microRNA expression and mesenchymal marker gene expression in glioblastoma. Neuro-oncology. 2012;14(9):1153-62.

4. Lv Z, Yang L. MiR-124 inhibits the growth of glioblastoma through the downregulation of SOS1. Molecular medicine reports. 2013;8(2):345-9.

5. Lu S-H, Jiang X-J, Xiao G-L, Liu D-Y, Yuan X-R. miR-124a restoration inhibits glioma cell proliferation and invasion by suppressing IQGAP1 and β-catenin. Oncology reports. 2014;32(5):2104-10.

6. Hayes J, Thygesen H, Tumilson C, Droop A, Boissinot M, Hughes TA, et al. Prediction of clinical outcome in glioblastoma using a biologically relevant nine-microRNA signature. Molecular oncology. 2015;9(3):704-14.

7. Cai J-J, Qi Z-X, Chen L-C, Yao Y, Gong Y, Mao Y. miR-124 suppresses the migration and invasion of glioma cells in vitro via Capn4. Oncology reports. 2016;35(1):284-90.

8. Li W, Huang H, Su J, Ji X, Zhang X, Zhang Z, et al. RETRACTED ARTICLE: miR-124 Acts as a Tumor Suppressor in Glioblastoma via the Inhibition of Signal Transducer and Activator of Transcription 3. Molecular neurobiology. 2017;54:2555-61.

9. Lv Z, Zhao Y. MiR-124 inhibits cell proliferation, invasion, and migration in glioma by targeting Smad2. International Journal of Clinical and Experimental Pathology. 2017;10(11):11369.

10. Stupak E, Veryaskina YA, Titov S, Achmerova L, Stupak V, Dolzhenko D, et al., editors. Studying the MicroRNA role as a survival predictor and revealing its part in malignancy level determination in patients with supratentorial gliomas of brain. AIP Conference Proceedings; 2017: AIP Publishing.

11. Ye X, Wei W, Zhang Z, He C, Yang R, Zhang J, et al. Identification of microRNAs associated with glioma diagnosis and prognosis. Oncotarget. 2017;8(16):26394.

12. Luo L, Chi H, Ling J. MiR-124-3p suppresses glioma aggressiveness via targeting of Fra-2. Pathology-Research and Practice. 2018;214(11):1825-34.

13. Zhang G, Chen L, Khan AA, Li B, Gu B, Lin F, et al. miRNA‐124‐3p/neuropilin‐1 (NRP‐1) axis plays an important role in mediating glioblastoma growth and angiogenesis. International journal of cancer. 2018;143(3):635-44.

14. Deng D, Luo K, Liu H, Nie X, Xue L, Wang R, et al. p62 acts as an oncogene and is targeted by miR-124-3p in glioma. Cancer Cell International. 2019;19:1-13.

15. Ma B, He X, Li Y, Zhang S, Hu W. MiR-124 inhibits malignant biological behaviors of glioma cells by targeting SDCBP. Int J Clin Exp Med. 2019;12(1):486-93.

16. Zhang Y, Chen J, Xue Q, Wang J, Zhao L, Han K, et al. Prognostic significance of MicroRNAs in glioma: a systematic review and meta‐analysis. BioMed Research International. 2019;2019(1):4015969.

17. Deng D, Wang L, Chen Y, Li B, Xue L, Shao N, et al. MicroRNA‐124‐3p regulates cell proliferation, invasion, apoptosis, and bioenergetics by targeting PIM1 in astrocytoma. Cancer science. 2016;107(7):899-907.

18. Wang S, Qi Y, Gao X, Qiu W, Liu Q, Guo X, et al. Hypoxia-induced lncRNA PDIA3P1 promotes mesenchymal transition via sponging of miR-124-3p in glioma. Cell Death & Disease. 2020;11(3):168.

19. Cai S, Shi C-J, Lu J-X, Wang Y-P, Yuan T, Wang X-P. miR‑124‑3p inhibits the viability and motility of glioblastoma multiforme by targeting RhoG. International Journal of Molecular Medicine. 2021;47(5):1-13.

20. Cheng H, Zhao H, Xiao X, Huang Q, Zeng W, Tian B, et al. Long non-coding RNA MALAT1 upregulates ZEB2 expression to promote malignant progression of glioma by attenuating miR-124. Molecular neurobiology. 2021;58:1006-16.

21. Stupak E, Veryaskina YA, Titov S, Achmerova L, Stupak V, Ivanov M, et al., editors. MicroRNA profiling in the malignant progression of gliomas. AIP Conference Proceedings; 2016: AIP Publishing.

22. Song Y, Bai L, Yan F, Chen C. Inhibition of EMMPRIN by microRNA‑124 suppresses the growth, invasion and tumorigenicity of gliomas. Experimental and Therapeutic Medicine. 2021;22(3):1-10.
